# Osteoporosis and alzheimer pathology: Role of cellular stress response and hormetic redox signaling in aging and bone remodeling

**DOI:** 10.3389/fphar.2014.00120

**Published:** 2014-06-10

**Authors:** Carolin Cornelius, Guido Koverech, Rosalia Crupi, Rosanna Di Paola, Angela Koverech, Francesca Lodato, Maria Scuto, Angela T. Salinaro, Salvatore Cuzzocrea, Edward J. Calabrese, Vittorio Calabrese

**Affiliations:** ^1^Department of Chemistry, University of CataniaCatania, Italy; ^2^Department of Clinical and Experimental Medicine and Pharmacology, School of MedicineMessina, Italy; ^3^Department of Biomedical Sciences, University of CataniaCatania, Italy; ^4^University of ManchesterManchester, UK; ^5^Environmental Health Sciences Division, School of Public Health, University of MassachusettsAmherst, MA, USA

**Keywords:** oxidative stress, redox status, Alzheimer’s disease, cellular stress response, hormesis, vitagenes

## Abstract

Alzheimer’s disease (AD) and osteoporosis are multifactorial progressive degenerative disorders. Increasing evidence shows that osteoporosis and hip fracture are common complication observed in AD patients, although the mechanisms underlying this association remain poorly understood. Reactive oxygen species (ROS) are emerging as intracellular redox signaling molecules involved in the regulation of bone metabolism, including receptor activator of nuclear factor-κB ligand-dependent osteoclast differentiation, but they also have cytotoxic effects that include lipoperoxidation and oxidative damage to proteins and DNA. ROS generation, which is implicated in the regulation of cellular stress response mechanisms, is an integrated, highly regulated, process under control of redox sensitive genes coding for redox proteins called vitagenes. Vitagenes, encoding for proteins such as heat shock proteins (Hsps) Hsp32, Hsp70, the thioredoxin, and the sirtuin protein, represent a systems controlling a complex network of intracellular signaling pathways relevant to life span and involved in the preservation of cellular homeostasis under stress conditions. Consistently, nutritional anti-oxidants have demonstrated their neuroprotective potential through a hormetic-dependent activation of vitagenes. The biological relevance of dose–response affects those strategies pointing to the optimal dosing to patients in the treatment of numerous diseases. Thus, the heat shock response has become an important hormetic target for novel cytoprotective strategies focusing on the pharmacological development of compounds capable of modulating stress response mechanisms. Here we discuss possible signaling mechanisms involved in the activation of vitagenes which, relevant to bone remodeling and through enhancement of cellular stress resistance provide a rationale to limit the deleterious consequences associated to homeostasis disruption with consequent impact on the aging process.

## INTRODUCTION

Mitochondrial medicine is emerging as powerful candidate for expanding anatomical and Mendelian biological concepts aimed to solve complexity in age-related diseases, aging and cancer (Di Domenico et al., 2010; Wallace, 2010, 2012, 2013). Interaction between structure and energy is a fundamental life prerequisite. This dualism in eukaryotic cell, was generated approximately two billion years ago after the symbiosis of a glycolytic progenitor, which evolved the nucleus–cytosolic compartment, and an oxidative progenitor evolving toward an ancient mitochondrion. Initially each proto-organism contained all the genes for an independent life. However, 1.2 billion years later, after subsequent genomic reorganizations and alternative rearrangements, was achieved a cellular arrangement in which the mitochondrial compartment became specialized in the generation of energy, while the nuclear and cytosolic compartment was functionally polarized toward structure. This final arrangement was the starting point for multicellularity fostering evolution in higher plants and animal kingdom, including humans. Notably, this original architecture comeback powerfully in our cells in all conditions where tumor initiation and promotion occur and the glycolytic tone of metabolic potential put in motion a process leading to the marginalization of energy transduction mechanisms, kicking off mitochondrial energy production in favor of a sustained high proliferative potential which underlie tumor progression (Calabrese et al., 2007c; Wallace, 2008; Perluigi et al., 2010; Bellia et al., 2011). In view of this, comprehension of aging mechanisms of aging and determinants of life span will contribute to decrease age-related morbidity and promote healthy aging. Over the last centuries, as a consequence of exogenous environmental factors and medical progress, average lifespan has increased, but maximal life span has not changed (Edrey et al., 2014; Willcox and Willcox, 2014). Consistent with this notion and relevant to longevity mechanisms, vitagenes by preserving cellular homeostasis during stressful conditions represent a functional network regulating life span. Vitagenes, are redox sensitive genes encoding for heat shock proteins (Hsps) Hsp32, Hsp70, for the thioredoxin and the sirtuin protein systems (Calabrese et al., 2008e; Bellia et al., 2009; Cornelius et al., 2013b). In this regard, nutritional anti-oxidants, have recently been demonstrated to be neuroprotective via hormetic pathways, including vitagenes. The hormetic feature of dose–response appears to quantitatively describe the limits of biological plasticity across phyla as well as at different levels of biological organization (cell, organ, and organism). Thus, pharmaceutical treatments for a wide range of human conditions can be based upon the hormetic biphasic dose response, as discovered within preclinical evaluations and then directly applied to human populations. The biological relevance of hormetic dose–response affects, ultimately, those strategies pointing to the optimal dosing to patients in the treatment of numerous diseases and, within this context, the heat shock response (HSR) can be considered an important hormetic target to design cytoprotective compounds able of inducing stress response mechanisms. Here we discuss signaling mechanisms activating vitagenes, which are relevant to bone remodeling which, through enhancement of stress resistance provide a rational therapeutic approach to limit the deleterious consequences associated to disruption of homeostasis with consequent impact on the aging process (Murshid et al., 2013; Saibil, 2013).

## ALZHEIMER’S DISEASE

Alzheimer’s disease (AD) is characterized by progressive deterioration of cognitive functions and stress (Calabrese et al., 2006d), with biochemical alterations consisting in the accumulation of amyloid-β (Aβ) protein in the form of senile plaques and intracellular neurofibrillary tangles, associated with hyperphosphorylated tau protein and neuronal cell depletion (Liu and Chan, 2014). Prevalence of AD rises exponentially with age, increasing drastically after 65 years. Sporadic AD, affecting more than 15 million people worldwide, is more common than familial AD (Richard and Brayne, 2014). Mild cognitive impairment (MCI) is regarded as a transition state between normal aging and dementia (Swomley et al., 2013). Importantly, almost one half of these individuals evolves to late onset AD (LOAD), accounting for about 60% of the total cases of dementia in USA and Western countries (Swomley et al., 2013). MCI, which can also be due to severe depression, extensive white matter pathology or severe vitamin B12 deficiency, is characterized by memory impairment with or without compromission in other single or multiple cognitive domains, in the absence of fulfillment of the standardized criteria for dementia (Swomley et al., 2013). The pathological hallmarks of AD are amyloid plaques, formed of Aβ peptide derived from the transmembrane amyloid precursor protein (APP; Siciliano et al., 2011), and neurofibrillary protein tangles, composed of hyperphosphorylated tau, in the temporal lobe and some other brain cortical regions associated with death of neuronal cells and synaptic depletion (Hardas et al., 2013). Given the heterogeneity of the etiologic factors underlying the pathophysiology of AD, although integrated approaches have been elaborated to explain its pathogenesis, such as Aβ aggregation, the precise definition of most critical factors determining the clinical onset and progression of the disease remains and elusive and a difficult task (Siciliano et al., 2011). Aβ peptide (Lodi et al., 2006; Wallace, 2013) has been shown to induce protein oxidation in both *in vitro* and *in vivo* studies (Hardas et al., 2013), and increasing evidence supports the role of free radical reactions in the pathogenesis of the disease. Consistent with this notion, it is demonstrated that these peptides form oligomers exerting neurotoxic effects by enhancing reactive oxygen species (ROS) level in the brain (Hardas et al., 2013). More specifically, Aβ oligomers directly generate H_2_O_2_ through: (i) a cupper-dependent superoxide dismutase-like activity (Fang et al., 2010), (ii) activation of NADPH-oxidase in astrocytes, (iii) modulation of mitochondrial ROS generation via regulation of activity of enzymes such as Aβ-binding alcohol dehydrogenase and α-ketoglutarate dehydrogenase (Borger et al., 2013). Increased proteotoxic and lipoperoxidative brain damage has been documented in subjects with MCI, compared to normal (Chico et al., 2013; Swomley et al., 2013; Cervellati et al., 2014). Thus, oxidative stress and disturbed protein metabolism and their interaction in a vicious cycle characterize AD as a protein misfolding disease, with protein clearance defects through the ubiquitin-proteasome system (Hong et al., 2014; Valasani et al., 2014). There is strong evidence, also, that APP may act as a trophic factor relevant to neurite outgrowth and synaptogenesis, as well as growth and cell proliferation (Abramov et al., 2009; Jiang et al., 2013; Bukanova et al., 2014; Dawkins and Small, 2014; Hughes et al., 2014). However, future research are required to fully clarify mechanisms of APP action (Dawkins and Small, 2014). In addition, identification of normal physiological functions of Aβ is an extremely important emerging issue, as many therapeutic strategies used for the treatment of AD point to prevent Aβ production or to increase Aβ clearance from the brain. Consistently, it cannot be, so far, ruled out that these strategies does not interfere with important physiological functions (Abramov et al., 2009; Kang et al., 2013; Bukanova et al., 2014; Mizoi et al., 2014; Ponnayyan Sulochana et al., 2014).

Among biochemical alterations occurring in AD pathology, deficit in choline esterase and overstimulation of NMDA receptors are the main target for current therapeutic approaches focusing on symptoms rather than more substantial eradication of the disease. Thus, to date there not drugs are available in the market capable of revert the disease, but only to influence the intensity of symptoms and the progression of the pathology (Ponnayyan Sulochana et al., 2014).

## CELLULAR STRESS RESPONSE AND THE VITAGENE NETWORK

It is known that low concentrations of ROS are able to induce the expression of anti-oxidant enzymes and other defense mechanisms. The basis to explain this phenomenon refers to the concept of hormesis (Calabrese et al., 2006b), which is a dose–response relationship in which a given substance is stimulatory at low dose while at high doses exerts inhibitory effects. Radical active species may be beneficial since they act as signals to enhance cellular defenses but become deleterious when present within a cells at high levels. ROS when in excess can over long-term disrupt redox homeostasis, cause oxidative stress, loss of molecular fidelity which underlie accumulation of unfolded or misfolded proteins in brain. Alzheimer’s, Parkinson’s, Huntington’s, amyotrophic lateral sclerosis, and Friedreich ataxia all belong to the so called “protein conformational diseases” affecting several thousands of aged people in all the world (Lodi et al., 2006). Unfolded protein response put in motion by chaperons actively rescue misfolded proteins, breaking up aggregates, and assisting the refolding process. Those proteins that cannot be rescued by refolding are, however, delivered to the proteasome by other chaperones and recycled (Lodi et al., 2006). In general, an unfolded protein response occur within conformational diseases characterized by dysfunctional aggregation of proteins accumulating in a non-native conformation. Under these physiopathological conditions, multiple metabolic derangements generally take place in association to excessive production of ROS and oxidative stress (Lodi et al., 2006).

**FIGURE 1:**
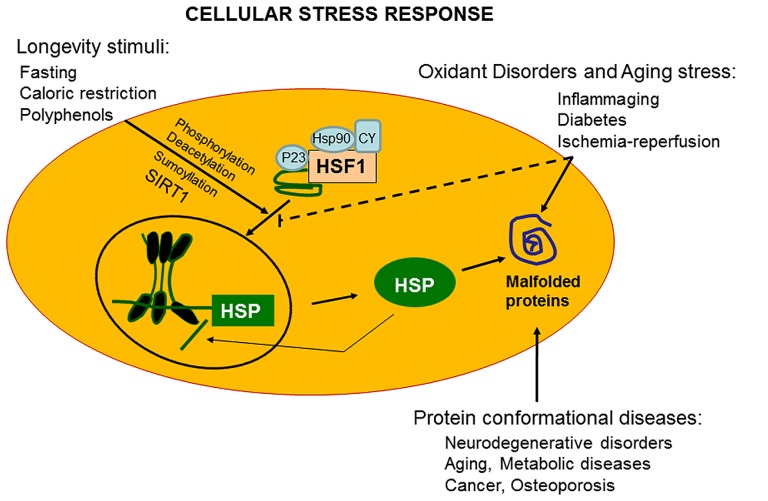
**Vitagenes and the pathway of cellular stress response.** Misfolded proteins cumulating in response to proteotoxic stresses trigger the cellular stress response. Hsps that are normally bound to HSF1, maintaining it in a repressed state before stress, are titrate away by damaged or misfolded proteins with resulting HSF-1 activation. Multi-step activation of HSF1 involves post-translational modifications, such as hyperphosphorylation, deacetylation, or sumoylation, which allow HSF1 to trimerize, translocate into the nucleus, and bind to heat shock elements (HSEs) in the promoter regions of its target Hsp genes. Nutritional anti-oxidants, are able to activate vitagenes, such as heme oxygenase, Hsp70, thioredoxin reductase and sirtuins which represent an integrated system for cellular stress tolerance. Activation of vitagene system, with up-regulation of HO-1, thioredoxin, GSH, and sirtuin, results in reduction of pro-oxidant conditions. During inflammaging, including aged-associated pathologies, such as Alzheimer’s disease and osteoporosis, a gradual decline in potency of the heat shock response occur and this may prevent repair of protein damage, leading to degeneration and cell death of critical parenchymal cells.

The ability of a cell to withstand stressful conditions is defined cellular stress response (**Figure 1**). This phenomenon includes the HSR and represents a highly conserved ancient mechanism of cytoprotection (Calabrese et al., 2006b; Lodi et al., 2006). Synthesis of Hsps, which includes protein chaperones, is fundamental for proper folding and repair of denatured proteins, thus promoting conditions favorable to cell survival that would otherwise result in apoptotic cell death (Calabrese et al., 2006c; Scapagnini et al., 2006). Chaperones promote cell survival by sequestering damaged, denatured proteins and inhibiting formation of aggregates. The response to formation of aggregates is coordinated by an elaborated regulatory system which, simultaneously to up-regulation of several chaperones and other survival-promoting proteins, silences most cellular highly energy demanding functions. For this reason, most chaperones are also defined Hsps in reference to heat shock as the prototypic form of cellular stress. Chaperones under normal conditions elicit multiple roles, promoting proteins or RNA transport and remodeling events in large protein complexes essential for the regulation of cell migration, differentiation, signaling, transcription, and proliferation. Cellular stress response consist of pro-survival pathways controlled by cytoprotective genes called vitagenes (Mancuso et al., 2007b), resulting in the production of molecules endowed with anti-oxidant and anti-apoptotic potential, such as Hsps, glutathione, bilirubin (BR), and carbon monoxide (CO; Calabrese et al., 2007a). Vitagenes include members of the Hsp family, such as heme oxygenase-1 and Hsp72, sirtuins and the thioredoxin/thioredoxin reductase system (Calabrese et al., 2008d). Increasing evidence suggests that the HSR promotes cytoprotective conditions in several human disease states, such as mild chronic inflammation, cancer, aging, and neurodegenerative diseases (Calabrese et al., 2007b, 2008e, 2011). Thus, an emerging interest is growing on the cytoprotective potential of the HSR as pharmacological agents capable of inducing the HSR are important candidate for novel anti-degenerative and anti-inflammatory therapeutic strategies (Abdul et al., 2006; Mancuso et al., 2007a; Calabrese et al., 2008b,g,j).

Transcriptional, translational, and post-translational regulation of cellular stress response occur with the intervention of heat shock transcription factors (HSFs) normally expressed and maintained in an inactive state (Calabrese and Maines, 2006; Perluigi et al., 2006; Calabrese et al., 2008f,h; Zhang et al., 2014). Post-translational regulation of HSFs is an emerging area of interest integrating the metabolic state of the cell with biology of stress, and hence controlling important complex aspects of proteome molecular fidelity and physiology with consequent impact on aging processes. The HSR is transcriptionally controlled through *cis*-acting sequences called heat shock elements (HSEs) present in multiple copies upstream of the Hsp genes. Unlike invertebrates showing a single HSF, vertebrates express multiple HSFs as for plants. Four mammalian members of HSF have been identified: HSF1, HSF2, HSF3, and HSF4, all recognizing and binding the HSE, formed by inverted repeats of nGAAn consensus sequences. Thus, all four HSFs have overlapping functions associated with distinct patterns of tissue-specific expression, and undergo multiple post-translational modifications and exhibiting various interacting protein partners. Up-regulation of Hsp synthesis in response to stress, besides heat shock, is also initiated by redox-dependent mechanisms, endogenous or exogenous, in both cases resulting in trimerization and DNA binding of HSF1. Relevant to transactivation process is the cysteine moiety which operates as critical redox switch leading to the HSF activation (Zhang et al., 2014). In particular, formation of an intermolecular disulfide bond between cysteine 36 and cysteine 103 within HSF1, trigger trimerization and subsequent DNA binding, whereas a disulfide intramolecular bond formation has inhibitory effects for the transactivating activity of HSF. Consistent with this notion, modulation of HSF-mediated gene regulation has an emerging pharmacologic potential, as small-molecule with the capability of activation or inhibition of HSFs, can exert important influence on aging, neurodegenerative processes and longevity mechanisms (Fujimoto et al., 2010; Zhang et al., 2011; Akerfelt et al., 2012; Westerheide et al., 2012). The regulatory domain of HSF has competence to sense heat shock, however, its induction is post-translationally controlled (Fujimoto et al., 2010; Akerfelt et al., 2012; Westerheide et al., 2012). In normal non-stressed cellular conditions, HSF1 is maintained inactive and in a monomeric state through interaction with Hsp90. Thus, pharmacological inhibition of Hsp90 results in a conversion of monomeric HSF1 in a trimerized HSF1 with DNA-binding competence (Calabrese et al., 2008c,i, 2009a,b,d). Trimerization, nuclear translocation and interaction with HSE in the DNA rapidly declines in cells soon after proteotoxic insult exposure, since HSF1 binding to the promoter occurs within seconds, reaching in about 1 min saturation with the contribution of other components such as a ribonucleoprotein complex containing the eukaryotic elongation factor 1A and a constitutive non-coding RNA, heat shock RNA-1 (HSR-1) endowed with heat-sensing capability (Calabrese et al., 2009c, 2010g,h). According to the most accredited model, in response to heat shock HSR-1 undergoes conformational changes facilitating HSF1 trimerization. Under this transactivating conditions, HSF1 is post-translationally modified through phosphorylation, sumoylation, and/or acetylation (Calabrese et al., 2010d, 2011). Increasing evidence suggests that phosphorylation and sumoylation of HSF1 is a phenomenon occurring rapidly after heat shock, while acetylation occurs with a delayed kinetics in coincidence of the attenuation phase of the cycle of HSF1 activation. In particular, acetylation of HSF1 is a process regulated by the ratio of acetylation/deacetylation controlled by p300–CBP (CREB-binding protein) and by the NAD^+^-dependent sirtuin, SIRT1. With respect to this, enhanced DNA-binding activity of HSF1 ensues after increased expression and activity of SIRT1, whereas acetylation of HSF1 consequent to down-regulation of SIRT1 reduces DNA-binding activity with no effect on the trimerization process (Calabrese et al., 2010d). Increasing evidence indicates the involvement of SIRT1 in caloric restriction and aging, as demonstrated in senescent cells where the age-dependent loss of SIRT1, associated to impairment in HSF1 activity, correlate with attenuation of the HSR and disruption of proteostatic mechanisms, thereby linking HSR to nutrition and aging (Calabrese et al., 2010e,f; Di Paola et al., 2011).

## Keap1/Nrf2/ARE BIOLOGY AND THE HEME OXYGENASE PATHWAY OF STRESS TOLERANCE

HSF1 is a central regulator in the gene expression of Hsps, however, in addition to this mechanism controlling vitagenes coordination, a response protecting against various electrophiles and oxidants operates integrating cytoprotection as “phase 2 response,” known also as “the electrophile counterattack response” (Zhang et al., 2011, 2014). Phase 2 response proteins include heme oxygenase 1, thioredoxin, and thioredoxin reductase, all of which can be up-regulated by the transcription factor Nrf2 (nuclear erythroid 2-related factor 2), similarly to other cytoprotective proteins, including NAD(P)H:quinone oxidoreductase 1 (NQO1), γ-glutamylcysteine synthetase, glutathione reductase, glutathione transferases (GST), UDP-glucuronosyltransferase, epoxide hydrolase, aldo-keto reductases, ferritin, and glutathione conjugate efflux pumps (Zhang et al., 2011, 2014). The elaborate network of Nrf2-dependent phase 2 response proteins enable eukaryotic cells to counteract the damaging effects associated to oxidants and electrophiles, agents primarily involved in the pathogenesis of neurodegenerative disorders, atherosclerosis, aging, and cancer (Zhang et al., 2011, 2014). A major factor operating in the control of the expression of these proteins is the Keap1/Nrf2/ARE system. Activation of gene expression requires that Nrf2, a basic leucine zipper transcription factor, binds in the upstream regulatory regions of these genes containing single or multiple copies of the anti-oxidant/electrophile response elements (ARE, EpRE). Nrf2 binding to the ARE occur in heterodimeric combinations with members of the small Maf family of transcription factors. Under normal conditions the pathway operates at basal levels controlled by a cytosolic protein with function of repressor, the Kelch-like ECH-associated protein 1 (Keap1). When Keap1 is bound to Nrf2, the latter is presented, by binding to the E3 ubiquitin ligase Cullin3–RING box1 (Cul3–Rbx1), to the ubiquitin system for proteasomal degradation. At least 10 distinct chemical classes of inducers of the Keap1/Nrf2/ARE pathway, are known and belong to: (i) Michael acceptors (olefins or acetylenes conjugated to electron-withdrawing groups); (ii) isothiocyanates; (iii) oxidizable diphenols, quinones, and phenylenediamines; (iv) thiocarbamates; (v) trivalent arsenicals; (vi) dithiolethiones; (vii) hydroperoxides; (viii) vicinal dimercaptans; (ix) heavy metals; and (x) polyenes. All these members share the common property of presenting chemical reactivity toward sulfhydryl groups through oxido-reduction, alkylation, or disulfide interchange mechanisms (Calabrese et al., 2010d, 2011; Zhang et al., 2011, 2014).

Heme oxygenase is a sensors of oxidative stress within cells modulating redox status and homeostasis. Heme oxygenase-1, an Hsp32, protects against oxidative damage converting pro-oxidant heme into biliverdin, free iron, and CO. With the intervention of the enzyme biliverdin reductase (BVR) biliverdin is then reduced by into BR, a linear anti-oxidant tetrapyrrole (Calabrese et al., 2010d). BR has been demonstrated efficiently buffer nitrosative stress, owing to its capability to bind and inactivate NO-derived RNS (Pennisi et al., 2011; Scapagnini et al., 2011). Two HO isoforms are known: an inducible isoform, HO-1 and HO-2 which is a constitutive enzyme (Calabrese and Maines, 2006). These isoforms share only 43% homology being products of different genes. HO-1 and HO-2 present important differences in cell and tissue regulation and distribution (Mancuso et al., 2006, 2008). Moreover, only in rat as third protein, HO-3 has been found to be retrotransposomal product of the HO-2 gene (pseudogene). In spite of playing the same enzymatic role, HO-1 and HO-2 have different protective function in tissues. HO-1 induction occurs as early response to oxidative insult resulting rapid transformation of heme into CO and BR. On the contrary, constitutive HO-2, regulates heme homeostasis a function associated within a cell with the sensing of intracellular levels of CO. HO-1, is induced in response to oxidative and nitrosative stress, heme, Aβ, dopamine analogs, H_2_O_2_, hyperoxia, UV light, heavy metals, prostaglandins, NO, peroxynitrite, Th1 cytokines, oxidized lipid products, and lipopolysaccharide, as well as growth factors. In all these conditions, the ARE contained in its promoter region, is central to its redox regulation (Alam and Cook, 2007; Calabrese et al., 2012b), recognizing two upstream enhancers, E1 and E2 where multiple ARE elements are contained. Consistently, polymorphisms in the lengths of GT repeats (Calabrese et al., 2011; Pennisi et al., 2011; Scapagnini et al., 2011) within the *ho-1* promoter is critical for the expression and functions of HO-1 in humans. Higher HO-1 activity is associated with the short-GT polymorphisms, a conditions thought be protective against oxidant disorders (e.g., coronary artery disease, atherosclerosis-linked conditions), whereas long GT sequences, coding for a relatively unstable, Z-conformational DNA, exhibits reduced transcriptional activity, as well as basal and stimulated HO-1 protein levels, as found in various malignant conditions (Calabrese et al., 2010d). Overexpression of HO-1 results in increased cGMP and bcl-2 levels in neurons, inactivation of p53, up-regulation of anti-oxidant proteins as well as ferritin which sequesters and inactivates free iron (Alam and Cook, 2007; Calabrese et al., 2012b). Although large consensus exists on the fact that increased HO-1 expression is a protective mechanism operating under oxidative stress conditions, however, there is evidence that HO-1 can be repressed during the same conditions, particularly during hypoxia. HO-1 repression under this condition is sustained by the heme-regulated transcription factors Bach1/Bach2 (Calabrese et al., 2010d, 2011) and it has been argued that this might be necessary to reduce the energy request due to heme degradation, to limit formation of CO and BR which can accumulate to toxic levels, and to increase heme availability for proper mitochondrial respiration (Calabrese et al., 2006c, 2008g). Neurodegeneration as a chronic inflammatory oxidative condition caused by Aβ metabolic disruption, makes HO-1 an important target for therapeutic anti-inflammatory strategies. HO-1 mRNA and protein expression have been documented to be increased in AD brain, in association with neurofibrillary tangles. This finding may have the biological meaning of a protective response in vulnerable neurons to transform pro-oxidant heme into BR and CO, whose anti-oxidant and anti-inflammatory nature has been ascertained. This opens new therapeutic avenue, as polyphenols contained in herbs and spices can represent potential drugs for the prevention and treatment of AD (Scapagnini et al., 2006; Calabrese et al., 2007a, 2008b). Epidemiological studies have demonstrated that curcumin is responsible for the significantly reduced (4.4-fold) prevalence of AD in India, compared to United States (Calabrese et al., 2007b, 2008b; Mancuso et al., 2007a), and curcumin given chronically in the diet to transgenic APPSw, a mouse model (Tg2576) of AD suppresses brain inflammatory and oxidative damage, an effect associated to inhibition of nuclear factor-κB (NF-κB) and efficient prevention of neuronal cell death (Calabrese et al., 2009b,d, 2010d,h; Gupta et al., 2011).

## HORMESIS

Hormesis is a dose–response phenomenon, characterized by a low-dose stimulation and a high-dose inhibition. The term hormesis was first introduced into the scientific literature in 1943 by Chester Southam and John Ehrlich, mycology researchers at the University of Idaho, who reported that low concentrations of extracts from the Red Cedar tree enhanced the metabolism of a number of fungal species. The term hormesis was derived from the Greek meaning to excite. Prior to the report of Southam and Ehrlich (1943), there was a substance history of reports in the biological literature on also demonstrating a similar biphasic dose response. It was first reported by Schulz who found that low concentrations of numerous disinfectants stimulated metabolism at low concentrations while being inhibitory at higher concentrations (Schulz, 1887, 1888). The research of Schulz was confirmed by other investigators and extended to other biological models and agents, with particular emphasis on bacteria, fungi, and plants over the next several decades. The phenomenon was initially referred to as the Arndt-Schulz Law and Hueppe’s Rule, with these terms being subsequently replaced by the term hormesis. The occurrence of hormetic dose responses from the time of Schulz up to approximately 1950 has been summarized in depth for both chemical and radiation induced hormesis (Calabrese and Baldwin, 2000a,b,c,d,e).

**FIGURE 2:**
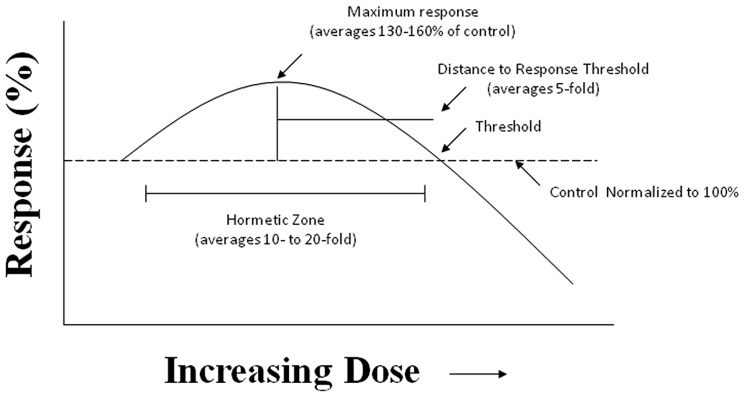
**The hormesis (biphasic) dose–response curve: generalized quantitative features.** The hormetic dose response is characterized by specific quantitative features concerning its amplitude and width. Based on more than 10,000 hormetic dose responses in various hormetic databases, approximately 80% have their maximum response less than twofold greater than the control. The strong majority of hormetic dose responses extend over approximately a 5- to 10-fold dose range immediately below the estimated response threshold. The hormetic dose response may occur either as a result of a direct stimulation or as an overcompensation response following an initial disruption in homeostasis, in both cases being the quantitative features similar. The hormetic response appears to quantitatively describe the limits of biological plasticity across phyla as well as at different levels of biological organization (cell, organ, and organism). The quantitative features of the hormetic dose response are also independent of mechanism. Pharmaceutical treatments for a wide range of human conditions are based upon the hormetic biphasic dose response as discovered within preclinical evaluations and then directly applied to human populations.

Despite the fact that the hormesis concept has considerable historical literature supporting its occurrence and reproducibility, it failed to gain traction within the biomedical community due to the fact that Schulz inappropriately claimed that he had discovered the explanatory principle of homeopathy, based on his biphasic dose response with yeast (Calabrese, 2005). This proclamation led to strong disputes with medically oriented researchers principally due to the long and intense conflict between homeopathy and traditional medicine. In fact, a considerable effort by leading pharmacologists of the Schulz era, such as Alfred J. Clark, devoted considerable effort to criticize Schulz and his biphasic dose response and to try to link it with high dilution homeopathy, in an effort to marginalize and discredit homeopathy (Calabrese, 2011). In addition to such historical antipathies, the concept of hormesis was also difficult to prove scientifically as it required a substantial number of doses, especially in the low dose zone, reasonably high statistical power, and biological model systems with generally low variability and the strong capacity for replication of findings. These factors are important since the low dose stimulation is modest, with most maximum stimulatory responses only being about 30–60% greater than the unexposed control group (**Figure 2**). Thus, trying to test hormetic hypotheses requires far more rigorous study designs and more resources than was usually the case in hazard assessment type investigations.

As a result of the historical conflicts between homeopathy and traditional medicine and the inherent challenges in testing hormetic hypotheses, the concept of hormesis never became well established as a scientific concept in throughout most of the twentieth century (Calabrese, 2008d). However, since the late 1970s there has been a multi-disciplinary-based resurgence of the hormesis concept, based largely on technical improvements in the capacity to measure at lower concentrations, advances in *in vitro* and high throughput research which has facilitated the capacity to test a wide range of concentrations in an efficient manner, the development of mechanistic understandings of biphasic dose responses especially at the receptor and cell signaling pathways levels and the dose response and mechanistic linkage of developments in the area of adaptive responses, including that of preconditioning with hormesis (Calabrese, 2010).

Within recent years substantial hormetic dose response data bases have been developed based on rigorous *a priori* entry and evaluative criteria providing documentation for the hormetic dose responses (Calabrese and Blain, 2005, 2009, 2011). These databases provide strong evidence that hormetic dose responses are independent of biological model, endpoint measured, the chemical class of the inducing agent, the level of biological organization (i.e., cell, organ, organism) and mechanism (Calabrese, 2013d). These databases have confirmed and far more firmly established the quantitative features of the hormetic dose response, revealing that it represents a modest stimulation, usually less than twice the value of the control group. It has also been established that the hormetic dose response is typically reported for highly integrated endpoints, especially those relating to cell proliferation, complex behaviors, adaptive/preconditioning responses, reproductive endpoints such as fecundity, cancer, mutation, cancer endpoints, and longevity/aging. Detailed evaluations of the pharmaceutical literature have also revealed that entire drug classes are based up the hormetic dose responses. For example, preclinical studies in the areas of anxiolytic drugs (Calabrese, 2008b), anti-seizure drugs (Calabrese, 2008e), memory enhancing agents (Calabrese, 2008a), drugs treating osteoporosis (Calabrese, 2008c), and others typically demonstrate hormetic dose responses. Since it is now believed that the modest hormetic stimulation is a measure of biological plasticity it suggests that the hormetic dose responses describes the degree to which pharmaceutical agents can enhance biological performance (Calabrese and Mattson, 2011; Calabrese, 2013a). It is important to note that during the entire twentieth century the biomedical and regulatory communities never validated the capacity of the threshold dose response to make accurate predictions below the threshold, that is, the area of the dose response where most people spend the majority of their time. This lead to a series of direct, head-to-head comparisons between the threshold, linear and hormetic dose response models using multiple large and independent dose response data sets with multiple models, endpoints and agents (Calabrese and Baldwin, 2001, 2003; Calabrese et al., 2006a, 2008a, 2010a). In each of these assessments, the hormetic dose response significantly out-performed the threshold and linear dose response models.

Major comprehensive evaluations of the biomedical literature have revealed that hormesis dose responses are commonly reported in essentially all areas of research including the immune system, tumor cell biology, neuroscience, including memory, stress, anxiety, seizure, pain, numerous degenerative diseases, wound healing and for a very broad range of receptor systems and peptides (Calabrese, 2013b). The applications of the hormesis dose response are therefore extensive, affecting drug discovery, drug development, and the design of the clinical trial. Furthermore, even areas such as preconditioning are based on the hormetic concept (Calabrese et al., 2010b,c, 2012a, 2013; Calabrese and Calabrese, 2013a,b; Krenz et al., 2013). This may be seen in numerous studies which have assessed a wide range of graded preconditioning doses with results being reflective of the biphasic-hormetic dose response. The historical assessment of the dose response has revealed that the dose response model that was once rejected by the biomedical community about a century ago is now the dose response model that has repeatedly outcompeted the traditional doses responses models in direct comparisons. It is the dose response model that has provided quantitative insight into the magnitude of biological plasticity and the theoretical basis for it. The hormetic dose response thus underlines the basis of pharmaceutical strategies aimed at enhancing biological performances in a broad range of scientific areas.

## BONE REMODELING, REDOX STATE, AND Aβ METABOLISM

Osteoporosis is a devastating disease having enormous health and economic impacts, particularly considering the global shift toward an aging population, characterized by a systemic and progressive skeletal pathology characterized by compromised bone mineral density and strength with the increased occurrence of fractures. Despite rapid progress in our understanding over recent years, patient morbidity and mortality resulting from this disease are still too high (Ono et al., 2014), and there is an urgent need for a proper assessment of the underlying mechanisms and the development of new treatment strategies to address this pathophysiological issue. Patients with AD show significantly increased risk of osteoporotic hip fractures. However, whether abnormal Aβ peptide (Aβ) deposition also occurs in osteoporosis and the relationship between Aβ and human osteoporosis remains an open, not elucidated, question (Li et al., 2014).

Amyloid-β peptide, one of the pathological hallmarks of AD, is a small (40–42 amino acids) proteolytic fragment of a glycosylated integral membrane cell surface receptor protein called APP and is encoded by a gene on human chromosome 21 (Liu and Chan, 2014; Richard and Brayne, 2014). Aβ has attracted much attention for its association with various pathologies (Stefanova et al., 2014). Besides AD, Aβ plays a crucial role in other important neurodegenerative diseases, such as Huntington’s, Parkinson’s, and prion disorders, as well as amyotrophic lateral sclerosis as well as type 2 diabetes and the most common age-related muscle disease of inclusion body myositis (Tóth et al., 2014). Thus, APP/Aβ seems to be associated with multiple degenerative disorders. Epidemiological studies showed that patients with AD had an increased risk of developing osteoporotic hip fractures even after considering the increased frequency of fallings in AD patients (Xia et al., 2013), suggesting one or more common denominators between both disorders. Nevertheless, an association between Aβ and human osteoporosis has not yet been clearly established, and also it has been inferred that Aβ may be of physiological importance for survival of cells (Li et al., 2014). Excessive Aβ aggregates and fibrillates to form amyloid plaques in the brain, thus leading to the exacerbation of AD pathology. Previous studies have identified a role for Aβ in the activation of osteoclasts through gene knockout experiments and use of the transgenic AD mouse model, Tg2576 (Li et al., 2014). However, whether a large amount of Aβ deposits also occur in osteoporotic bone tissues and the role human Aβ may play on OC activation remain unclear. In addition to having an activation effect on osteoclasts, Aβ may accumulate abnormally in osteoporotic bone and play an important pathogenic role. A close relationship between Aβ and osteoporosis is shown across species from rodent to human, as demonstrated in different clinical conditions in patient samples as well as in various animal model and cell cultures. AD and osteoporotic hip fractures often coexist during aging. Platelets have been shown to be the primary source (90%) of Aβ in human blood with plasma Aβ levels fluctuating over time among individuals (Roher et al., 2009). Besides human plasma and cerebrospinal fluid, soluble Aβ is also a component of human urine in Alzheimer’s. Despite this level of knowledge surrounding APP and Aβ expression in many tissues, reports on the expression and distribution of Aβ in bone tissues and osteocytes remain an emerging evidence. Aβ deposition has been found on the endosteal and periosteal surfaces of adult rat ulnae (Li et al., 2014). Notably, occurrence of Aβ and APP abnormal accumulation in different tissues supports the hypothesis that Aβ diseases may be a systemic disease, suggesting that these malfolded proteins may either be produced locally in diverse organs or may originate from a common circulating precursor. Consistent with this notion, abnormal Aβ and APP burden has been detected in osteoporotic bone tissues from both human and rat OVX models, where Aβ42 was identified mainly in the membrane and cytoplasm of osteocytes and extracellular matrix, while APP largely found in the membrane of osteocytes. Despite increasing research efforts, still the mechanism underlying the accumulation of Aβ and APP in osteocytes in osteoporotic bones remains elusive. One possible source for Aβ deposition in bone may be blood, where Aβ increases during senescence (Li et al., 2014). In addition to this, secretion of Aβ by mature osteoblasts has been documented, in agreement with the finding of Aβ42 and APP formation in osteoblasts from both human and OVX rats osteoporotic bone. In these conditions, APP has been found to be able of suppressing osteoblast differentiation, associated with osteoporotic alterations (Xia et al., 2013). Given that osteoblast is the precursor of the osteocyte, it is conceivable that the deposition of Aβ and APP in osteocytes can be consequence of secretion by osteoblasts during both osteogenic differentiation and aging processes. In turn, accumulating Aβ may promote apoptotic process in osteocytes, likewise in neurons thus determining bone loss and osteoporosis (Cui et al., 2011). An interesting and controversial question concerns why the abundant presence of proteins of Aβ abnormal metabolism in the bone of osteoporotic patients did not cause them to develop brain degeneration, such as AD. A number of explanations may exist to provide a possible rationale. First, bone is a very special organ, with limited blood supply, with most of the osteocytes embedded in the matrix without direct contact with blood. In these conditions, release of Aβ into the blood stream is not an easy process. Second, the blood–brain-barrier (BBB) permeability can be of extreme importance for the prevention of Aβ invasion into the brain tissues, as uptake of peripheral Aβ by the brain is not a normal occurrence without BBB compromission (Jefferies et al., 2013). Lastly, both osteoporosis and AD are multifactorial diseases with complex etiology and pathogenesis (Rachner et al., 2011). Aβ deposits are present in several tissues, which indicates that the protein may originate as product of local metabolism in various organs or, similarly to other amyloidoses, can derive from a circulating precursor common to all these pathophysiological conditions. However, further studies are necessary to explore these dynamics and understand the underlying mechanisms. Abnormal Aβ deposition in osteoporotic bone tissues and its potent enhancement effect on osteoclast differentiation and activation, is already clearly demonstrated suggesting an important role for Aβ in the pathogenesis of osteoporosis (Li et al., 2014). This is of great clinical significance for providing novel insights into the tight link between Aβ and human osteoporosis, thus revealing a potential mechanism underlying altered bone mineral density by Aβ abnormal metabolism. Clearly, however, further work is required to elucidate the exact mechanisms through which Aβ regulates osteoporosis signaling. These research efforts may eventually lead to a promising future discovery of a new etiology for osteoporosis, and prompt healthcare professionals and researchers to develop innovative anti-bone-resorptive therapeutic agents and strategies, particularly those designed by targeting Aβ, to efficiently minimize deleterious consequences associated with bone homeostasis disruption. In line with these evidence, since a biomarker is a traceable substance indicating changes in expression or metabolism of a given protein which correlates with the risk or progression of a disease, as consequence, Aβ may be a novel and promising candidate biomarker for drug targeting and characterization of osteoporotic therapeutic approaches in the future (Osorio et al., 2014).

**FIGURE 3:**
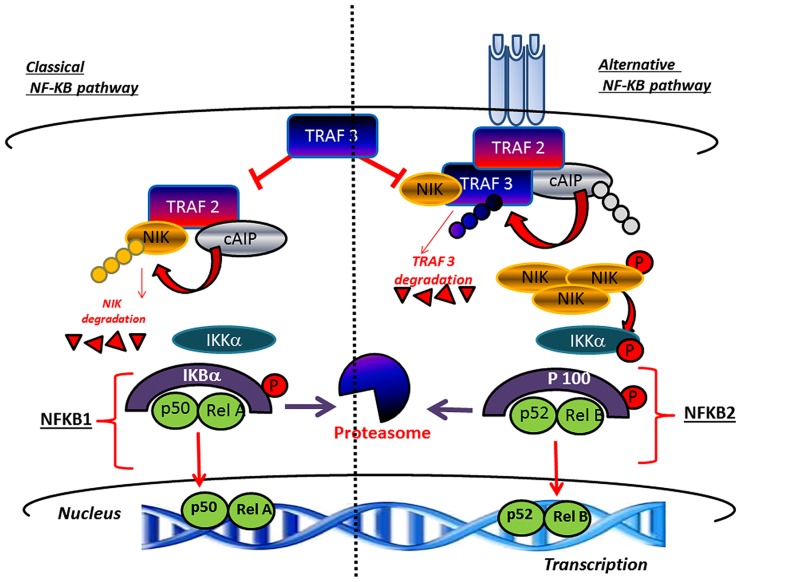
**Canonical and non-canonical pathway leading to the activation of NF-κB.** TRAF3 inhibits activation of the classical NFκB pathway A high level of TRAF3 interferes with the recruitment of TRAF2 to the receptor. In parallel, TRAF3 induces NIK degradation and consequently inhibits the activation of the alternative arm of NFκB. The activation of canonical pathway results in the phosphorylation of IκBαα by the IKK complex, leading to its ubiquitylation and subsequent degradation by the proteasome. The RelA/p50 complex is free to translocate to the nucleus to activate the transcription of target genes. The non-canonical pathway results in the NIK stabilization. In response to receptor crosslinking, TRAFs and cIAP1/2 are recruited to the receptor, where cIAP1/2 ubiquitinates TRAF2 and TRAF3 and stimulates their degradation. Accumulated NIK activates IKKα , which in turn phosphorylates p100, leading to p100 processing to p52, which can lead to the activation of p52-RelB that target distinct κB element and induce the transcription of target genes.

Bone tissue undergoes, throughout life, a continuous renewal through a process called bone remodeling, which is controlled by the activity of osteoclasts mediating bone resorption and parallel activity of osteoblasts which mediate bone formation (Vacek et al., 2013). Any disturbance in the balanced formation and resorption process, which can be linked to hormone disequilibrium or aging decreases bone mass and result in bone pathologies, such as osteoporosis leading to increased vulnerability to fractures. Within this context, the receptor activator of NF-κB ligand (RANKL) appears to be an important factor underlying osteoporosis pathogenesis for its critical role played in osteoclast differentiation and activation (Park et al., 2014). For this reason, inhibition of RANKL represents an innovative therapeutic target for controlling osteoclastogenesis (Park et al., 2011). Notably, an important role in bone remodeling is played by alternative or non-canonical NF-κB pathway, which mediates activation of the p52/RelB NF-κB complex, thus regulating various biological processes. This pathway differently from IκBα degradation in the canonical mechanism, consists of processing of p100 a NF-κB2 precursor protein,. In this context a central role is played by NF-κB-inducing kinase (NIK), a component of the non-canonical NF-κB pathway and a downstream kinase, IKKα (inhibitor of NF-κB kinase) which operate with integrated functions promoting induction of phosphorylation-dependent ubiquitination of p100. Under normal conditions, NIK is processed by a tumor necrosis factor (TNF) receptor-associated factor-3 (TRAF3)-dependent E3 ubiquitin ligase. After signals mediated by a subset of TNF receptor superfamily members, TRAF3 is degradated and NIK is stabilized leading to non-canonical activation of NF-κB (Sun, 2012; **Figure 3**). Accordingly, the inhibitory role of p100, in both basal and stimulated osteoclastogenesis in bone formation as well as resorption has been clearly demonstrated (Soysa et al., 2010). In the alternative NF-κB pathway p52 derived from p100 through NIK, binding of p52 and RelB induces effects on osteoclast biology (Soysa et al., 2010). However, to date, the precise physiologic importance of alternative NF-κB in bone biology, is not completely elucidated. Furthermore, the currently known intracellular signaling pathways activated after receptor binding of RANKL include the nuclear factor of activated T cells (Piva et al., 2009), mitogen-activated protein kinases (MAPKs), TRAFs, c-Jun N-terminal kinases (JNKs), and ROS (Kaunitz and Yamaguchi, 2008; Kanzaki et al., 2013). In addition, NF-κB is a transcription factor, which pleiotropically regulate osteoclast formation, function, and survival (Piva et al., 2009). Deletion of both NF-κB p50 and p52 subunits is associated to osteopetrosis as consequence of osteoclast absence and, in addition, NF-κB is central for the differentiation of RANK-expressing osteoclasts into osteoclasts TRAP^+^ induced by osteoclastogenic cytokines. This explain the inhibitory effect on osteoclast formation induced by prevention of NF-κB activation (Piva et al., 2009; Augustine and Horwitz, 2013).

Reactive oxygen species act as intracellular signaling molecules involved in the regulation of RANKL-dependent osteoclast differentiation, but they also have cytotoxic effects that include peroxidation of lipids and oxidative damage to proteins and DNA. Taking into account the relationship between Nrf2 and osteoclastogenesis, stimulation of osteoclast precursors (mouse primary peritoneal macrophages and RAW 264.7 cells) with RANKL results in the up-regulation of Keap1, a negative regulator of Nrf2, with decreased Nrf2/Keap1 ratio, and down-regulation of cytoprotective enzymes, such as heme oxygenase-1 and γ-glutamylcysteine synthetase (Kanzaki et al., 2013). On the other hand, Nrf2 overexpression results in up-regulation of the expression of cytoprotective enzymes, associated with decrease in ROS levels, tartrate-resistant acid phosphatase-positive multinucleated cell number, as well as osteoclast differentiation, and attenuation of bone destruction, as found both *in vitro* and *in vivo* models (Kanzaki et al., 2013). Consistent with this line of evidence, overexpression of Keap1 or RNAi-induced knock-down of Nrf2 resulted in effects opposite to those obtained by stimulation of Nrf2-dependent DNA binding activity (Kanzaki et al., 2013). The precise mechanisms by which stimulation with RANKL reduces Nrf2 is not currently known. It is known Keap1 has highly reactive thiol groups in its structure and that oxidation of this domain leads to significant changes in the conformation of Keap1, resulting in dissociation from Nrf2 and stimulation of nuclear Nrf2-dependent DNA binding activity (Kanzaki et al., 2013). In addition, Nrf2 (see previous section) autoregulates its own expression (Calabrese et al., 2010d; Zhang et al., 2011, 2014). Taken together, this evidence implies that an increase in ROS levels induced by stimulation with RANKL may up-regulate Nrf2. It has also been reported that Nrf2 regulates Keap1 by controlling its transcription (Calabrese et al., 2010d; Zhang et al., 2014). Change of stability of Nrf2 mRNA or decrease of translation by miRNA can modulate RANKL-dependent Nrf2 down-regulation. Also, Bach1, an inhibitor of Nrf2 binding to the ARE, could participate to this mechanism, as indicated by attenuated osteoclastogenesis found in Bach1 knock-out mice (Kanzaki et al., 2013). However, although extensive investigations will be required to clarify the exact regulatory mechanisms linking Nrf2 to stimulation with RANKL, it is clearly proven that Keap1/Nrf2 axis regulates RANKL-dependent osteoclastogenesis through redox-modulation of intracellular ROS signaling and expression of cytoprotective enzymes. This raises the exciting possibility that the Keap1–Nrf2 axis may be a therapeutic target for the treatment of bone destructive disease.

## CONCLUSION

Comprehensive evaluation of the biomedical literature has pinpointed the role of hormetic dose responses as reported in essentially all areas of research, including the immune system, tumor cell biology, neuroscience, drugs treating osteoporosis, as well as wound healing and a broad range of receptor systems and peptides (Calabrese, 2008c, 2013b). Applications of the hormesis dose response are therefore extensive, affecting drug discovery, drug development, and the design of the clinical trial. It is now conceivably expected that the hormetic concept is at the core of attempts leading to enhanced development of biological shields via processes such as preconditioning which can help to improve the quality normal living, the aging process and to reduce the impact of various types of degenerative diseases. AD is a chronically progressive disorder, in which neurodegenerative damage progresses for many years before clinical onset (Calabrese et al., 2006d; Liu and Chan, 2014; Richard and Brayne, 2014). Osteoporosis is a progressive degenerative disorder, also recognizing a multifactorial origin that is characterized by bone deterioration resulting in fragility and fractures. The medical and social and economic effects of osteoporosis, given the increasingly aging population, in particular osteoporosis of postmenopausal nature, will growth drastically. A better knowledge of bone biology with particular regard to the functional relationship between bone-formation by osteoblast activity and bone-resorption by osteoclasts with a better definition of the underlying signaling network has unraveled novel therapeutic targets. Osteoporosis and hip fracture are commonly observed complications seen in patients with AD. Although the mechanisms underlying this association remain poorly understood, emerging evidence supports the view that AD risk genes may also be a risk factor for osteoporosis, and that AD and osteoporosis may share conserved oxidative stress-driven pathogenic mechanisms. Consistent with this notion, using transgenic mice expressing an AD-associated mutant form of APP, known as APPswe, to explore the effects of mutant APP on bone remodeling, significant defects in bone formation associated with reduced differentiation of bone marrow stromal cells into osteoblasts in *ex vivo* culture experiments, were found in these mice compared with wild-type controls. Treatment with *N*-acetylcysteine ameliorated the impact of APPswe on bone marrow stromal cell differentiation, and increased bone volume in these transgenic mice (Woodman, 2013). Thus, ROS may be a conserved mechanism underlying APPswe-induced neurodegenerative and osteoporotic pathological alterations. Bone remodeling is a process of continuous formation and resorption occurring in specific areas of the matrix. Novel therapeutic strategies have been developed focused on the inhibition of excessive bone resorption and promotion of bone formation process. Due to novel drugs available, using the most recent knowledge of bone-cell biology, we have increased our therapeutic intervention to osteoporotic patients with more individualized lines of treatment. Accordingly, basic research can greatly contribute to the identification of specific pathways that can be effectively targeted by novel compounds able to treat and possibly reverse osteoporosis, particularly that occur in already chronically severed patients, such as in neurodegenerative disorders.

## Conflict of Interest Statement

The authors declare that the research was conducted in the absence of any commercial or financial relationships that could be construed as a potential conflict of interest.
